# Psychological Evaluation of a Residential Children’s Burns Camp Programme: A Ten-Year Evaluation

**DOI:** 10.3390/ebj4030033

**Published:** 2023-09-21

**Authors:** Alison M. Thomlinson, Niamh R. Farrell, Mamta Shah, Sarah L. Gaskell

**Affiliations:** Paediatric Burns Service, Manchester University NHS Foundation Trust, Manchester M13 9WL, UK; niamh.farrell@mft.nhs.uk (N.R.F.); mamta.shah@mft.nhs.uk (M.S.); sarah.gaskell@mft.nhs.uk (S.L.G.)

**Keywords:** burns camp, evaluation, burn rehabilitation

## Abstract

Residential burns camp programmes provide help and support to children with burn injuries by providing activities designed to build their confidence and self-esteem. Our regional burns service has been running camps for over 20 years and evaluation is an important part of assessing their effectiveness. In this study, we report both qualitative and quantitative data from 10 consecutive years of burns camps. Qualitative feedback was gathered using Likert scales and free-response questionnaires at the end of camp and six weeks post-camp. Three quantitative outcome measures, the Paediatric Quality of Life Inventory v4, the Children’s Revised Impact of Event Scale (CRIES8) and the Satisfaction with Appearance scale (SWAP), were completed before and six weeks after camp. Both children and their parents/carers reported that attending the burns camp was helpful for them/their child; meeting other children with burn injuries and developing confidence and self-esteem were cited as reasons. Parents/carers also reported improvements in their child’s physical and psychological functioning post-camp in some years, although these results were not as clear as the qualitative findings. These findings confirm the importance of providing burns camp programmes for children with burn injuries as part of their post-burn rehabilitation.

## 1. Introduction

Experiencing a burn injury as a child can be a traumatic experience, and the process of recovery is difficult with a vigorous aftercare routine including painful dressing changes, physiotherapy, and specialist pressure garments for scar management [1]. Some children may also need psychological support after their burn injury to help them cope with the emotional impact of the injury, the demands of their treatment regime and coming to terms with and adjusting to a changed appearance, including dealing with questions and curiosity from others [2,3]. 

Burns camp programmes provide support for children with fun and confidence-building activities that help them adjust to the changes they have experienced following their burn injury. The first burn camps were held in America in the early 1980s [4,5] and, by 2010, burns camps were being held in over 60 countries worldwide [6]; that number continues to grow to this day [7,8]). The positive effects of attending a burns camp have been reported in the literature for over 20 years, with most studies focusing on qualitative methods to assess the impact of attending a burns camp. Cox et al. reported improved body image in teenagers [9] and several groups found campers reported increased self-confidence, increased acceptance of scars and altered body image following the camp. They also found developing social skills and sharing experiences of burn injury were important to camp participants [10,11,12,13]. More recently, a long-term retrospective follow-up study from Australia found that the benefits of the camp programme were long term, particularly for those who developed friendships that continue outside the camp experience [14]. 

Some studies have used quantitative methods to try to capture changes in teenagers’ perception of self-esteem and body image after attending a burn camp programme. Biggs et al. [15], Arnoldo et al. [16] and Bakker et al. [13] found either no change or only small changes in self-esteem using the Rosenberg self-esteem tool [17]. Tropez-Arceneaux et al. also found no significant short-term changes using the Rosenberg self-esteem tool [7]. Bakker et al. [13] and Armstrong-James et al. [18] both found significant short-term improvements in campers’ satisfaction with their appearance using the Satisfaction with Appearance scale [19].

Burns rehabilitation camps have become an integral part of burns aftercare and in the UK, the National Burn Care Standards [20] stipulate that Burn Care Services should provide access to a Burns Camp/Club for children and young people up to the age of 25. Our regional paediatric burns service has been running children’s burn camps since 1993. It was originally a weekend break for a small number of children and staff but by 2000, this had expanded into two, age-specific, week-long residential camps (along with additional activity days and family weekends) over the course of a calendar year. The camp programme focuses on fostering an affirming environment for children to explore social connections and build self-esteem through physical activity, mindfully informed routines, and reflective activities. Evaluation data have been collected at each camp. In 2007, Gaskell reported on the challenges of evaluating burns camps, especially with quantitative outcome measures [10]. In 2008, the Psychosocial Special Interest Group of the British Burn Association (BBA) reviewed the outcome measures available for assessing well-being after a burn injury at the time and found no validated screening tools for paediatric burn populations. The BBA recommended using a group of four measures: Paediatric Quality of Life (PedsQL), post-traumatic stress (CRIES8), the Satisfaction with Appearance scale and a pain/discomfort measure to assess both child well-being and parent/carer coping [21]. Whilst these measures are not burn specific, they were agreed as being the best tools available for assessing post-burn well-being at the time and were trialled by several UK paediatric burns services [22]. As a result, the quantitative measures included in the camp evaluation questionnaires were changed to include three of the measures (the assessment for pain was omitted). 

Here, we report the evaluation findings from ten consecutive years’ longitudinal evaluation of each of our two, age-specific, weeklong residential burns camps using both qualitative feedback questionnaires and these quantitative outcome measures. It is the first burns camp programme to report the use of these quality of life and emotional impact outcome measures. The results of each year’s evaluation were used to improve the camp programmes in subsequent years for the benefit of our patients.

## 2. Materials and Methods

### 2.1. Participants

Access to the camp was through professional referral by any member of the multidisciplinary burn care team. Places at both the younger and older children’s camps were also offered to the paediatric burns service in an adjacent centre, typically 2 and 4 places, respectively. The campers were placed in the two camps depending on their age: the younger children’s camp for children aged between 5 and 10 years and the older camp for children between 10 and 16 years. Ten year olds were allocated to either the younger or older children’s camp as appropriate for the individual child, based on both the clinical referral and parent/carer advice. Children were invited to camp by telephone conversation with a parent/carer; verbal acceptance was followed up with an application form to be completed by children and their parents/carers. In 2010, 2017 and 2019, two teenagers taking part in an exchange programme with an American burn camp programme joined the older children’s camp but did not take part in the evaluation. 

### 2.2. Burn Camp Leaders 

The campers were supported by a 2:1 ratio of camp leaders. The camp leaders were recruited from the multidisciplinary burn care teams at both burns services, including doctors, nurses, physiotherapists, occupational therapists, clinical psychologists and play specialists; fire fighters from the local Fire and Rescue Service and burns camp volunteers, including burns survivors who were now adults.

### 2.3. Procedure

The BBA Psychosocial Special Interest Group-recommended outcome measures were trialled at the older children’s camp (10–16 years) in 2009 then used for both younger children’s (5–10 years) and older children’s camps between 2010 and 2019. Qualitative information was also collected for each camp. Children aged 8 and above and their parents/carers were asked to complete the same age-appropriate outcome measure questionnaire before and six weeks post-camp, the pre-camp questionnaires were sent out with the invitation letters to each parent/carer and child as appropriate. All children were asked to complete, a simple one-page questionnaire (developed and piloted at camps prior to 2010) at the end of the camp to gather qualitative data; they were reassured handwriting and spelling did not matter and help with writing was provided by camp leaders, if needed. Children and parents/carers were then asked to complete a similar one page questionnaire as part of the six week follow up. Camp leaders were also asked to complete a post-camp evaluation questionnaire. 

### 2.4. Qualitative Tools

The questionnaire included simple 7-point Likert scales and free-response questions. At the end of the camp, children were asked if they had/had not enjoyed the camp, if the camp had helped them (2010–2014) and if they would recommend the burns camp to someone who had a burn injury (2015–2019). The scale ranged from one (did not enjoy/is not helpful/would not recommend) through to seven (enjoyed a lot/is very helpful/would definitely recommend). The free-response questions then asked what they had/had not enjoyed, how the camp had helped them and why they would/would not recommend the camp to someone with burn injuries. Finally, they were asked how the camp programme could be improved. The follow up questionnaire, six weeks post-camp, asked everyone if attending a burns camp helped children with burn injuries and how the camp could be improved. Parents/carers and camp leaders were asked what they felt children gained by attending a burns camp. The responses to these qualitative tools were grouped into themes by the lead author.

As the end of camp evaluation questionnaires were completed anonymously, to encourage completion, it was not possible to directly compare them with the post-camp questionnaires or look at whether there were any differences in the responses between the children who went on to complete the follow-up and those who did not.

### 2.5. Quantitative Measures

Three outcome measures were included. 

The Paediatric Quality of Life Inventory (PedsQL) v4 measure [23] consists of separate age-specific parent and child reports for quality of life and emotional well-being. This 23 item scale is designed to measure the core dimensions of health as defined by the World Health Organisation and covers domains of cognitive function, communication, worry, daily activities and family relationships; each item is scored on a 5-point scale from 0 (never a problem) to 5 (always a problem), the overall score for each subscale on version 4 ranges from 0 to 100. Lower scores are indicative of greater difficulties within each domain. Parents/carers of all children completed a proxy report for both physical and psychosocial functioning while children aged 8 and above completed a self-report of the same measures; there is no child report for 5–7 year olds. Parents/carers also completed self-reports for health-related quality of life and family functioning to assess parental coping. Upton et al. have shown this measure is reliable and valid for populations of UK children [24]. 

The Children’s Revised Impact of Event Scale (CRIES8) [25] is a child-friendly self-reporting measure of anxiety/distress for children aged 8 and above. Items are scored as 0 (not at all), 1 (rarely), 3 (sometimes) and 5 (often); the total score ranges from 0 to 40. Scores of >17 indicate a child has risk factors for post-traumatic stress disorder. It has been shown to positively correlate with measures of anxiety and depression [26] and children diagnosed with post-traumatic stress disorder (PTSD) have been shown to score significantly higher than children without PTSD [27]. Any child reporting scores of >17 was referred to the burns clinical psychologist for follow up. 

The Satisfaction with Appearance scale (SWAP) [19] was used with the older children’s camp for young people aged 13 and over. It is a measure of post-burn body image designed to identify patients who develop a negative body image following a burn injury; the 14 item scale measures both satisfaction with appearance and the social-behavioural impact of burns scars and has been used previously with paediatric burns patients [13,18]. Higher scores indicate dissatisfaction with appearance and a poorer body image.

### 2.6. Statistical Analysis

Descriptive statistics are reported for both the qualitative Likert scales and the quantitative outcome measures. Likert scale data are expressed as the median number of responses for each point on the scale with the interquartile range (IQR) providing a measure of the spread. Paired pre- and post-camp data from the outcome measures were analysed after each camp. The change in the quantitative scores is presented as median and IQR for each year separately as the results of each year’s evaluation were used to improve the camp programmes in subsequent years and the small sample sizes.

## 3. Results

During the study period of 2010–2019, between 14 and 19 children attended the younger children’s camp each year and between 21 and 28 attended the older children’s camp. Each camp comprised of first time and returning campers. In total, 400 places at the camp were taken up over the ten-year period: 158 at the younger children’s camp and 242 at the older children’s camp. The median age range of participants at the younger children’s camp was 6.0–10.0 years; and at the older children’s camp, it was 12.0–14.0 years (Table 1). 

### 3.1. Qualitative Responses

#### 3.1.1. End of Camp Evaluation

The end of camp evaluation was completed by all the children attending the camp; over 70% of the children attending each camp reported enjoying it a lot, or twelve of the twenty camps the figure was over 90%. A summary of the Likert scale responses to the questions “How much has camp helped you?” and “Would you recommend the camp to someone else who has been burned?” is provided in Table 2, a median response of 1 or more is given in bold type. Whilst the most common score given was 7 with a median of 10 responses (younger) and 12 responses (older), respectively, there is a spread of responses, particularly for the older children, as to whether the camp helped them. Over the whole ten-year period, only 7 children reported scores of less than 4, four from the younger camp and three from the older camp. A variety of reasons for the low score were given; two children thought the camp had not helped at all, although one of them also asked to be invited back and two reported not liking the activities. Three gave positive comments including “It helped a lot”, “I know it’s fun and not boring” and “To know I’m not alone”. The vast majority of those who attended the camp would recommend a burns camp to someone else with burns. Again, the most common score given was 7 with a median of 13 responses (younger) and 22 responses (older).

The free text responses were grouped into themes. The themes of *Meeting children with burn injuries* and *Developing confidence and self-esteem* were repeated in all ten years at both of the camps. A theme of *Camp is great fun* was seen every year after the question change in 2015. A selection of quotes from these three themes can be found in Table 3. 

Other frequent themes appeared in at least 8 of the 10 years of this study. *Learning to cope with a burn injury* was seen as important, examples of campers responses include “I have to massage my burn, it hurt but it should get better” (older child, 2011) and “I think because you can do full activities and you know burns don’t restrict you” (younger child, 2016). *Developing friendships and social skills* was another frequently repeated theme with responses such as ”Meeting new people and making friends” (older child, 2017) and “Learned to be kind and helpful (younger child, 2014).

#### 3.1.2. Follow up Evaluation

The follow up qualitative questionnaires were completed by children aged 8 and over and parents/carers of all children six weeks after the camp. The response rate varied from camp to camp, as can be seen in Table 2, with a slightly higher median response rate for parents/carers than children and a better median response rate for participants of the older children’s camp. A better response rate was achieved in the years a reminder was sent to families. The Likert scale summary (Table 2) clearly shows respondents continued to think that attending the burns camp was beneficial for a child with burn injuries six weeks later. Most children thought “Coming to burns camp helps people who have been burned”, 7 was the most common score, with medians of 6 (YC) and 13 (OC) responses, respectively. Only 1 child from each camp reported a score of less than 4, both in 2010, but no reason was given in either case. The parents/carers thought the camp helped their child and they gained a lot by attending. Again, the most common score was 7 for both questions. Three parents/carers gave a score below 4, one with a younger child (2016) who had language difficulties and two with older children (2010 and 2013) gave no reason.

Free responses were again grouped into themes; themes appearing every year are presented in Table 4 with a few illustrative quotes. *Meeting children with burn injuries* is seen as an important part of helping children come to terms with their injury by both younger and older children and their parents/carers. This help could be anything from just seeing another child with scars to receiving encouragement from other children to manage their own treatment regime, e.g., using their creams and wearing pressure garments. The theme of *Developing confidence and self-esteem* was reported by older children, their parents/carers and camp leaders after every camp. Children often felt more confident about showing their scars after attending the burns camp and became more comfortable wearing t-shirts, shorts and/or swimming costumes (authors’ observations). Comments were also made on the theme of the *Camp programme* by both children and their parents/carers, with frequent comments about the range of activities on offer and the request to make the camp longer. Parents/carers felt the camp helped their children *Develop friendships, social skills and peer support*; the theme appeared after 8 of the 10 older children’s camps and 7 of the 9 younger children’s camps (post-camp data from families were not available from the younger children’s camp in 2010). The type of comments made by the children and their parents/carers were similar after every camp: “I made friends” (younger child, 2012); “You can offer people support and be there for each other” (older child, 2015); “Friendships and interaction skills” (parent/carer, 2013) and “Peer support is awesome” (parent/carer, 2017). Older children often gave more detailed responses than younger children. One exception was comments relating to the weather which were only made after a wet week. 

Camp leaders also reported that attending a burns camp helped children *Develop friendships, social skills and peer support;* the theme appeared after every older children’s camp and 9 of the 10 younger children’s camps; comments included “The children made new friends, supported each other and gained new skills and experiences” (2011) and “Making new friends without feeling self-conscious” (2016). The themes of the *Camp programme* and the importance of the *Teamwork between Camp Leaders* in delivering the range of camp activities and providing the rehabilitative support to the children appeared after every camp. Camp programme comments included “The range of activities is great”(2013, 2018), “The swimming was excellent, and the centre had everything we needed” (2014) and “Using ‘Rose, Bud, Thorn’ as a wind down activity worked well”(2017); examples of leaders teamwork responses included “We had a really good staff team who worked well together and put a lot of effort into making the campers feel safe and supported” (2017) and “Having previous campers now as adult leaders has an obvious positive message, I thought their contribution was invaluable this year” (2019). In several years, leaders at the younger children’s camp gave comments on the theme of *Learning to cope with a burn injury*, examples include “I think some of the children had a very positive camp experience which helped them come to terms with their injuries/scars” (2013) and “Confidence to manage and talk about their scars” (2018). 

### 3.2. Quantitative Responses

The response rate for the post-camp outcome measure questionnaire was the same or slightly higher than the free-response questionnaire, occasionally families would skip the Likert scale and free-response page. The outcome measures data were more variable than the qualitative responses; the pre- and post-camp data are often similar with only small differences between the scores. Table 5 presents the data as the change between the pre-camp and post-camp scores, for each year, with the number of responses, the median and IQR for each measure given. An improved quality of life measure is denoted by a positive median value (higher score post-camp indicates a reported improvement in that quality of life domain) whereas improved trauma and satisfaction with appearance scores are denoted by a negative median (a lower score post-camp).

The change observed is variable between measures and between years. Improvements (median of 4 or more) in quality of life measures were reported by parents/carers of younger children in 2011, 2013, 2015, 2016, 2018 and 2019, but only by parents/carers of older children in 2010, 2011 and 2013. Parents/carers of older children also reported a deterioration in health related quality of life and family functioning in 2014. Children reported improved quality of life after attending the burns camp in 2011, 2013, 2016, 2017 and 2018 (younger) and 2013 (older). Younger children exhibited fewer trauma concerns (median of −4 or less) following the camp in 2017, 2018 and 2019 but higher concerns in 2011 and 2013; the number of respondents in these two years is very low and, with one exception, each child scored 17 or more both pre- and post-camp so were known to be experiencing trauma symptoms. Older children exhibited less change on the trauma measure. The teenagers completing the appearance satisfaction scale showed both improvement (2010 and 2017) and deterioration (2012 and 2013) in their appearance concerns as well as six years with little reported change.

## 4. Discussion

Experiencing a significant burn injury is a traumatic event, with children often spending a prolonged period of time in hospital away from family and peers. Transition back into normal routines and social spaces involves adjusting to both temporary (dressings and pressure garments) and long-lasting (e.g., scarring) changes to appearance [1,2,3]. Burns camps aim to provide a safe space for children to face the challenges of recovery in a fun, supportive and affirming environment. 

Our qualitative findings show children who attended a weeklong burns camp do indeed report positive benefits at the end of the camp programme that were maintained at the post-camp follow up six weeks later. Whilst a spread of responses can be seen on the Likert scales, the majority are at the top two points of the scale, showing that both children and their parents/carers felt strongly that the burns camp programme helped them/their child in a number of ways. 

Consistent themes were observed from the free-response comments in all ten years of the evaluation covered by this report. Meeting other children with burn injuries shows children they are not the only one with burn scars and facilitates the development of peer support networks which are often maintained outside the camp environment. We have several cohorts of campers who are now in their 20s and 30s who still maintain these friendships and support each other on an ongoing basis (personal communication). Some have gone on to train as volunteer burns camp leaders and are now an invaluable part of the programme, providing unique support for children based on their lived experience. This highlights the value of creating a supportive environment for children to expand their support networks through validation and connection to a shared experience of a potentially isolating and life-altering injury [10]. In this context, children and their parents/carers reported that the camp experience restored confidence and facilitated adjustment to the life changes brought about by the injury experience with coping strategies learned from each other. The responses received from parents/carers post-camp echoed those of their children; with developing confidence, self-esteem and independence as well as being able to talk to other children with burn injuries cited as ways in which attending a burns camp helped their child. This is consistent with other qualitative reports in the burn’s literature [11,12,13,14]. This feedback also highlighted the importance of having a good team of burn camp leaders who work well together to support the range of activities provided at the camp and provide any psychosocial support required. While the burn camp programme does not include any specific psychological intervention measures, the burns service clinical psychologist is embedded in the burn camp leader team and any child triggering concerns is followed up after the camp with appropriate targeted intervention. 

In 1997, Marion Doctor commented, “While it is commonly observed that young people are positively affected by the camp experience, quantification of that differentiation has proven somewhat elusive” [28]. Gaskell in 2007 [10] and a recent review by Kornhaber et al. [29] both highlighted the inconsistencies between positive qualitative data and non-significant changes seen with quantitative data. As Gaskell reported, from 1999 the outcome measures completed by participants of the older children’s camp included tools for general and emotional well-being, social relationships and self-esteem, none provided any demonstrable change [10]. These measures were designed for a general population and had not been standardised on burn-injured populations. By changing the outcome measures used in our pre-camp and follow-up questionnaires to those evaluated by the BBA Psychosocial Special Interest Group [21,22] we hoped to improve our quantitative data set and demonstrate quantifiable benefits of attending a burns camp. Feedback to clinicians from patients who had attended a burns camp prior to 2010 cited improvements in appearance concerns after the camp; including the Satisfaction with Appearance scale for older children provided a means to measure this. Improvements were observed on the parent/carer quality of life measure for both younger and older children on occasions, but not consistently over the period of study. However, unlike Armstrong-James [18], who reported a significant improvement in participants’ satisfaction with their appearance three months post-camp, this study only found a positive change in two of the ten years at the six-week follow up. For these two years (2010 and 2017), the pre-camp and follow up scores reported by both studies were very similar with a similar number of respondents (Armstrong-James study *n* = 11, this study *n* = 9). Unfortunately amalgamating the data into a single data set to increase the sample size would not be a valid comparison because each camp is a discrete intervention with a slightly different programme, different cohort of children and in some year’s very different external variables (e.g., weather) which all have an impact. The hypothesis that the decrease in health-related quality of life reported by parents/carers in some years maybe due to children’s improved confidence post-camp is also suggested as a possible explanation for the lower Strengths and Difficulties Questionnaire score reported by parents in the Armstrong-James study [18]. As previously discussed, the three outcome measures chosen are not burns specific and so the measures may not be sensitive enough to pick up changes in small cohorts of burns patients. As and when burn-specific outcome measures, validated for the paediatric population become available, these will be incorporated into the evaluation of our burn camp programme. 

It is also worth noting the observed increase in the median age of patients attending the younger children’s camp from 2013; informal feedback suggests this is likely due to a growing sense amongst parents/carers of younger children that 4 nights is too long to be away from home. Consequently, the burns camp day activities we have offered to patients since returning to face-to-face activities in 2022 have focused on the younger age range (5–12 years) with the residential camp programme offered to 8–16 year olds. The age spread was more consistent at the older children’s camp over the years, with a median age of 13 in seven of the ten years.

## 5. Conclusions

Over a period of ten consecutive years, children attending a regional residential burns camp consistently reported the benefits of the programme to include meeting other burn-injured children and developing confidence and self-esteem. This was echoed by both their parents/carers and the camp leaders. As with many other studies, the quantitative measures did not reliably match the qualitative data throughout but did provide corroboration in some years.

## Figures and Tables

**Table 1:** Number and median age, in years, of children attending a residential burn camp between 2010 and 2019.

	Younger Children’s Camp				Older Children’s Camp			
Year	Total Number	Girls	Boys	Median Age	Total Number	Girls	Boys	Median Age
2010	17	9	8	6.5	23	11	12	13.0
2011	15	9	6	6.0	21	9	12	13.0
2012	15	9	6	7.0	21	12	9	13.0
2013	18	9	9	8.0	24	13	11	13.0
2014	14	7	7	7.5	22	12	10	12.0
2015	19	8	11	8.0	24	16	8	13.0
2016	16	8	8	8.0	25	18	7	12.0
2017	15	9	6	9.0	28	17	11	14.0
2018	15	8	7	9.0	27	14	13	13.0
2019	14	8	6	10.0	27	18	9	13.0

**Table 2:** Summary of Likert scale responses provided by (a) children on the last night of camp, (b) children, aged 8 and above, six weeks post-camp and (c) parents/carers six weeks post-camp for both the younger children’s (YC) and older children’s (OC) camps. The results are given as the median (25th percentile, 75th percentile) for those reporting each score from 2010 to 2019; *n* = number of years and the interquartile range provides a measure of the spread of the data. The response rate for each question is also shown. The score scale ranges from 1, not at all, through to 7, very much and a median response of 1 or more is given in bold type. Post-camp data were not available for the younger children’s camp in 2010.

		Likert Scale Score							Response Rate (%)
		1 (not at all)	2	3	4	5	6	7 (very much)	
**(a) End of Camp—all children**									
**How much as coming to camp helped you?**	YC *n* = 5	0 (0, 0.5)	0 (0, 0.5)	0 (0, 1)	**1 (0.5, 2)**	0 (0, 1)	**2 (1.5, 2)**	**10 (8, 11.5)**	100.0
	OC *n* = 4	0 (0, 0.75)	0 (0, 0)	0.5 (0, 1)	**1 (0.25, 1.8)**	**3.5 (1.5, 4.8)**	**2.5 (2, 4.4)**	**12 (8.8, 17)**	100.0
Would you recommed the Camp to someone else who has been burned?	YC *n* = 5	0 (0, 0)	0 (0, 0)	0 (0, 0)	0 (0, 1.5)	**1 (0, 2)**	**1 (0, 2)**	**13 (11, 15)**	100.0
	OC *n* = 6	0 (0, 0)	0 (0, 0)	0 (0, 0)	**1 (0, 1.3)**	0 (0, 1.3)	**1.5 (0, 2.3)**	**22 (20, 23)**	100.0
**(b) Post Camp—children aged 8 and above**									
How much do you think coming to Burns Camp helps people who have been burned?	YC *n* = 9	0 (0, 0)	0 (0, 0)	0 (0, 0)	0 (0, 0)	0 (0, 0)	0 (0, 0)	**6 (4.5, 7)**	60.0
	OC *n* = 10	0 (0, 0)	0 (0, 0)	0 (0, 0)	0 (0, 0.25)	0 (0, 1)	**1.5 (0, 2.3)**	**13 (10,18)**	69.5
**(c) Post Camp—** **parent/carer**									
How much do you think that coming to camp has helped your child?	YC *n* = 9	0 (0, 0)	0 (0, 0)	0 (0, 0)	0 (0, 1)	0 (0, 1)	**1 (0, 2)**	**3 (1, 7.5)**	67.0
	OC *n* = 10	0 (0, 0)	0 (0, 0)	0 (0, 0)	0 (0, 0.25)	**1 (0, 1.3)**	**2 (0.75, 2)**	**13 (9.5, 17)**	71.0
How much do you think your child gained from coming to camp?	YC *n* = 9	0 (0, 0)	0 (0, 0)	0 (0, 0)	0 (0, 1)	0 (0, 1)	**1 (0.5, 2)**	**6 (1, 8)**	67.0
	OC *n* = 10	0 (0, 0)	0 (0, 0)	0 (0, 0)	0 (0, 0.25)	**0.5 (0, 1)**	**2 (0.75, 2.3)**	**13 (8.8, 16)**	71.0

**Table 3:** Repeated qualitative response themes given by children at the end of the camp. The themes of *meeting children with burn injuries* and *developing confidence and self-esteem* appeared every year from (a) 2010 and 2019 and *camp is great fun* every year from (b) 2015–2019 in response to an additional question added to the questionnaire in 2015.

Themes	Illustrative Quotes	
	Younger Children	Older Children
(a) 2010–2019		
Meeting children with burn injuries	− Knowing that other people have also been burnt and knowing what they went through (2010) − You get to meet other children with burns (2015) − They know they are not the only one with a burn (2016) − You can talk to other people about their burns (2019)	− I have been able to meet people that suffer from burns and seen that you can still carry out ambitions and goals to whatever extent you want, having a burn doesn’t stop you (2010) − You know you’re not alone and you’re not the only one that has scars (2012) − It helps you meet other people with burns and also helps you understand and accept your own burns (2015) − It helps you talk to people who have been through the same thing as you (2019)
Develop confidence and self-esteem	− Camp has helped me a lot, it gave me confidence (2012) − Helped me be independent (2014) − They will take lots and lots of care of you and it will build your confidence in different activities (2016) − It builds your confidence (2017)	− Made me feel more confident about my scars and myself (2009) − I am more confident showing my burns (2012) − It helps you be more confident with your burn (2014) − It has boosted my confidence about my burn as sometimes I’m not confident about it (2019)
(b) 2015–2019		
Camp is great fun	− It’s fun and the activities are good (2015) − I’ve enjoyed it and it’s fun (2016) − It is so much fun and you make a lot of amazing memories (2019)	− I would recommend the camp to someone else because it is really fun (2015) − Camp is a great and wonderful experience which every child who has been burned should try (2016) − I would recommend it to someone else who had been burned because camp is really fun, exciting (2018)

**Table 4:** Repeated qualitative response themes given by children, aged 8 and above, and their parent/carers six-week post-camp; several themes appeared in the evaluation of both the camps every year. In 2010, the six-week follow up was not completed after the younger children’s camp.

Themes	Illustrative Quotes		
	Younger Children	Older Children	Parent/Carer
Meeting children with burn injuries	− To just see other people burnt and not just feeling that only you have burns (2012) − I think it helps because we can talk to people who have experienced the same things (2014) − It makes you feel like you are not the only person that has been burned (2016) − It helps to have people around you who have scars like you, you can talk about it if you want to (2018)	− Makes me see that I am not the only one who has been burned (2010) − Helps you realise you’re not alone and there are people out there who know how you feel and what you’re going through and they can give you advice (2012) − You meet people who have gone through what you went through, and it helps by talking to them (2016) − Makes us feel that we are not on our own and there’s other people like us and we can still move on with our life no matter what (2018)	− Meeting other children with burns has helped [Name] realise she’s not the only one (2011) − He learnt that others have injuries and is less embarrassed, he is less aggressive with family members (2012) − Meeting other children who had similar experiences and injuries was incredibly important in [Name]’s progress (2016) − Meeting other children who had experienced the same thing, showing him, he was not alone has helped with anxiety and coming to terms with his accident (2017)
Developing confidence and self- esteem	−	− It gives you the confidence to show your scars without feeling intimidated or embarrassed (2011) − I think burns camp improves our confidence (2015) − I’ve got a lot more confidence (2017) − It allows people to feel more confident in themselves and helps them feel less self-conscious (2019)	− Confidence to not be embarrassed by her injuries in public (2010) − Helped with his confidence and how to cope with bullies at school (2013) − [Name] gained confidence and seems to be mixing more with other kids rather than playing on her own (2016) − I noticed a difference in [Name]’s confidence immediately. The child we left to go to camp was anxious, the child we picked up a week later was confident and beaming from the experiences she had had (2018)
Camp programme	− Lots of fun, good games (2012) − I got an ice cream, went climbing and got to ride my favourite horse (2016) − Stay for longer and not wait a year to go again (2019)	− Make it longer (2010) − I enjoyed all the sport especially the swimming (2014) − It’s a personal preference but more archery sessions please (2018)	− There are loads of well organised activities (2011) − The activities are incredible (2014) − The activities and doing things in the camp (2018)

**Table 5:** Change between pre-camp and post-camp outcome measure scores in each year expressed as median (25th percentile, 7th percentile); *n* = number of paired responses. Outcome measure data are not available for the younger children’s camp in 2010.

	2010	2011	2012	2013	2014	2015	2016	2017	2018	2019
**(a) Younger Children**										
QL parent—physical		*n = 12*	*n = 12*	*n = 12*	*n = 11*	*n = 13*	*n = 9*	*n = 13*	*n = 9*	*n = 8*
		0 (−0.8, 27.2)	−3 (−13.8, 2.5)	4.5 (−1, 7.8)	3 (0, 9.5)	0 (−6, 3)	6 (0, 18)	0 (−7, 8)	0 (0, 0)	0 (−3.8, 9.8)
QL parent—psychosocial		*n = 12*	*n = 12*	*n = 12*	*n = 11*	*n = 13*	*n = 9*	*n = 13*	*n = 9*	*n = 8*
		0 (−10, 14.8)	1.5 (−7.2, 5.5)	1.5 (−7, 8.2)	0 (−7, 4)	5 (−7, 25)	10 (1, 13)	−3 (−7, 13)	3 (−3, 9)	1 (−4.5, 5)
Health Related QL		*n = 12*	*n = 12*	*n = 12*	*n = 11*	*n = 13*	*n = 9*	*n = 13*	*n = 9*	*n = 8*
		4.5 (−6.5, 13)	2 (−6.8, 9.2)	−0.5 (−10.8, 7.2)	−1 (−6.5, 2)	−2 (−8, −1)	1 (−1, 7)	2 (−2, 10)	8 (0, 12)	4.5 (−3.2, 18.5)
Family Functioning QL		*n = 12*	*n = 12*	*n = 12*	*n = 11*	*n = 12*	*n = 8*	*n = 13*	*n = 9*	*n = 8*
		−2.5 (−9.2, 12.2)	0 (−4.5, 4.5)	1.5 (−13.8, 7.8)	0 (−14, 0)	−3.5 (−15.2, 0)	8 (0, 22)	0 (−3, 0)	10 (3, 22)	0 (−8.5, 12)
QL camper—physical		*n = 3*	*n = 4*	*n = 4*	*n = 5*	*n = 8*	*n = 6*	*n = 8*	*n = 8*	*n = 6*
		6 (−2, 12)	2 (−6.2, 6.2)	6 (2.2, 10)	0 (−6, 0)	0 (−0.8, 1.5)	−1.5 (−3, 0)	6 (3, 12.2)	0 (−1.8, 0.8)	1.5 (−2.2, 5.2)
QL camper—psychosocial		*n = 3*	*n = 4*	*n = 4*	*n = 5*	*n = 8*	*n = 6*	*n = 8*	*n = 8*	*n = 6*
		3 (−8.5, 11.5)	−2 (−7, 12.8)	9 (5.2, 13.2)	0 (−2, 0)	1 (−4.2, 10.2)	5 (1.2, 5.8)	0.5 (−1.2, 4.2)	6.5 (−0.2, 21.2)	3.5 (−0.2, 5)
Revised Impact of Events		*n = 3*	*n = 4*	*n = 4*	*n = 5*	*n = 7*	*n = 6*	*n = 8*	*n = 8*	*n = 6*
		8 (−3.5, 12.5)	−1.5 (−3.8, 2.5)	5.5 (4.8, 6.2)	0 (−3, 0)	0 (−1.5, 2)	0 (−6.8, 0.8)	−5.5 (−7.2, 4.8)	−4 (−9.5, 2.5)	−7.5 (−13.5, −1.5)
** (b) Older Children **										
QL parent—physical	*n = 15*	*n = 12*	*n = 12*	*n = 12*	*n = 11*	*n = 17*	*n = 18*	*n = 20*	*n = 19*	*n = 19*
	0 (−1.5, 10.5)	0 (−1.5, 3.8)	−3 (−10, 3.8)	0 (−3.2, 7)	−3 (−8, 1.5)	0 (−12, 0)	0 (0, 7.5)	0 (−4.5, 0)	0 (−3, 0)	0 (0, 4.5)
QL parent—psychosocial	*n = 15*	*n = 12*	*n = 12*	*n = 12*	*n = 11*	*n = 17*	*n = 18*	*n = 21*	*n = 19*	*n = 19*
	7 (0.5, 12.5)	7.5 (−1.2, 17)	−5 (−13, 6.2)	1 (−6.2, 7.2)	−3 (−15, 2)	0 (−8, 8)	2.5 (0, 9)	0 (−7, 9)	3 (−3, 11.5)	0 (−6.5, 5)
Health Related QL	*n = 15*	*n = 11*	*n = 12*	*n = 11*	*n = 11*	*n = 17*	*n = 17*	*n = 20*	*n = 18*	*n = 19*
	0 (−9, 11)	3 (−6.5, 7.5)	−2 (−8.2, 1.5)	0 (0, 6.5)	−6 (−10.5, −0.5)	0 (−5, 3)	0 (−9, 10)	0 (−4.2, 4.2)	0 (−4.8, 0)	0 (−4.5, 1)
Family Functioning QL	*n = 15*	*n = 11*	*n = 12*	*n = 11*	*n = 11*	*n = 17*	*n = 18*	*n = 20*	*n = 17*	*n = 19*
	0 (−11, 5.5)	0 (−6, 3)	−1.5 (−9, 0)	0 (−14, 2)	−7 (−30, 0)	0 (−31, 0)	0 (−9, 3)	0 (−0.8, 0)	0 (0, 0)	0 (0, 0)
QL camper—physical	*n = 15*	*n = 12*	*n = 12*	*n = 12*	*n = 11*	*n = 17*	*n = 18*	*n = 20*	*n = 18*	*n = 19*
	0 (−8, 0)	0 (−11.2, 4.5)	0 (−7.5, 3.8)	0 (−0.8, 4.5)	0 (0, 3.5)	0 (−7, 0)	0 (−5.2, 0)	0 (0, 0.8)	0 (−3, 3)	0 (−1.5, 3)
QL camper—psychosocial	*n = 15*	*n = 12*	*n = 11*	*n = 12*	*n = 11*	*n = 17*	*n = 18*	*n = 20*	*n = 18*	*n = 19*
	−3 (−19.5, 2.5)	−2.5 (−5, 2.8)	−5 (−11, 5)	4 (−5.2, 7.8)	1 (−11, 7)	0 (−5, 7)	0 (−1.8, 2)	2 (0, 5.2)	0 (−2.8, 5.5)	0 (−2.5, 5.5)
Revised Impact of Events	*n = 15*	*n = 12*	*n = 12*	*n = 12*	*n = 11*	*n = 16*	*n = 18*	*n = 20*	*n = 18*	*n = 19*
	−3 (−5, 4)	0 (−10, 2.5)	−3 (−6.2, 3)	−0.5 (−1, 0.8)	−2 (−8, 2)	−0.5 (−4, 0)	0 (−1.8, 5.2)	0 (−5.5, 0.5)	0 (−8.5, 1)	0 (−2, 1)
Satisfaction with Appearance	*n* = 10	*n* = 8	*n* = 6	*n* = 3	*n* = 4	*n* = 8	*n* = 8	*n* = 8	*n* = 6	*n* = 14
	−8.5 (−18.2, 2)	−2 (−6.8, 2.5)	4.5 (0.2, 7.2)	6 (4, 6.5)	3 (−8.5, 4.8)	0.5 (−3.2, 5.5)	0 (−7.5, 15.5)	−7 (−12.2, 0)	1 (−2, 2.5)	−2 (−18, 3.5)

## Data Availability

Restrictions apply to the availability of the data as it sits within confidential patient records.
